# Ectomycorrhizal community associated with *Cedrus deodara* in four urban forests of Nantong in East China

**DOI:** 10.3389/fpls.2023.1226720

**Published:** 2023-08-30

**Authors:** Zhugui Wen, Chunyan Lin, Xiaoming Xu, Simiao Ma, Yue Peng, Yue Sun, Boping Tang, Liang Shi

**Affiliations:** ^1^ Jiangsu Key Laboratory for Bioresources of Saline Soils, Jiangsu Synthetic Innovation Center for Coastal Bio-agriculture, Jiangsu Provincial Key Laboratory of Coastal Wetland Bioresources and Environmental Protection, School of Wetlands, Yancheng Teachers University, Yancheng, China; ^2^ College of Life Sciences, Nanjing Agricultural University, Nanjing, China

**Keywords:** ectomycorrhizal (EM) fungi, *Cedrus deodara*, community composition, soil properties, seedlings growth

## Abstract

Ectomycorrhizal (ECM) fungi play fundamental roles in host plant growth and terrestrial ecosystems. *Cedrus deodara* is cultivated in several regions in China, has high ecological, economic and medicinal value, for its afforestation and providing timber and wood oil. Here, we investigated ECM colonization status of four urban *C. deodara* forests in Nantong, East China. We also characterized soil spore banks by conducting bioassay experiments using soils collected from these forests. In total, we identified 19 ECM fungal species, of which 13 species were found in mature forests and 9 species were identified in bioassay experiments, with only 3 species shared. Soil pH and available P content had significant effects on species occurrence in both mature trees and bioassay seedlings on local scales. ECM communities clearly (A = 0.391, p = 0.006) separated mature forests from spore banks. Thelephoracae was the richest family we detected associated with *C. deodara*, while *Trichophaea sp*. was the most dominant in mature forests, and *Wilcoxina sp*. was dominant in spore banks. ECM richness affected the growth of bioassay seedlings, especially after inoculation with 2 ECM species, promoting root growth, significantly (F = 3.028, p = 0.050), but it had no effects on shoots (F = 1.778, p = 0.177). No effect of inoculation rate was found on seedlings growth. To conserve this important tree species, the ECM fungi that are associated with it should be considered.

## Introduction

As associations form between specialized soil fungi (Ectomycorrhizal fungi, ECM fungi) and the roots of plants, ectomycorrhizae play an important role in the growth and survival of many tree species in natural forest environments (Smith and Read, 2008). Host trees depend largely on ECM fungi for nutrients absorption and cycling. In a stable forest system, tens of hundreds of ECM fungi coexist with their hosts (Miyamoto et al., 2014), and their mycelia or spores are ubiquitous in forest soil, especially in Pinaceae and Fagaceae (Marcel et al., 2015; Brundrett and Tedersoo, 2018; Corrales et al., 2018). The ECM fungal community is an essential component of forest ecosystems (Smith and Read, 2008; Bennett et al., 2017), and correspondingly, environmental factors can also affect their structures.

Recent studies have shown that deterministic and stochastic processes can affect ECM fungal community structures. For example, host phylogeny, as the most important deterministic process, has been confirmed in the Betulaceae, Salicaceae, and Fagaceae species (Tedersoo et al., 2012; Wu et al., 2018; Wang et al., 2021a), even though some studies (Miyamoto et al., 2015; Glassman et al., 2017) have shown that the host influence is minor. Similarly, some abiotic environmental factors, such as other deterministic processes, including soil and climatic variables, have been discussed (Nickel et al., 2017; Castanão et al., 2018; Rosinger et al., 2018; Wen et al., 2018; Arraiano-Castilho et al., 2021; Zhang et al., 2021; Gong et al., 2022). However, fungal characteristics (such as different species, spore size, and yield) (Peay et al., 2012; Kivlin et al., 2014) and the geographic distance (Glassman et al., 2015; Wen et al., 2015) stochastically contribute to the determination of the ECM fungal community assembly. In particular, the soil spore banks of some ECM fungi can, however, maintain their infectivity under disturbance and form associations with the seedlings. Thus, in recent years, soil spore bank communities have received increasing attention, especially under adverse conditions (Chen et al., 2015; Murata et al., 2017; Wen et al., 2018; Wen et al., 2022). Many studies (Matsuda et al., 2009; Obase et al., 2009; Arai et al., 2017; Wang et al., 2021b; Zhang et al., 2021) have shown that the diversity and community of ECM fungi are of great significance for the establishment of coastal forests. However, no study has focused on the ECM fungi associated with *Cedrus deodara* in this particular ecosystem in East China.

*Cedrus deodara* belongs to the family Pinaceae and is widely distributed in southern Tibet, India, and Afghanistan and listed in the International Union for Conservation of Nature (IUCN) Red List (IUCN v. 3.1). *C. deodara* is one of the most useful tree species as almost every part has good uses (Nishtha and Thakur, 2021). There is only one type of this species of *Cedrus*, and it is cultivated in several regions in China, with its high ecological, economic, and medicinal value recognized due to its role in afforestation and providing timber and wood oil. It is recorded in the dictionary of Chinese Crude Drugs as an effective herbal drug for many indications, such as wind–cold–dampness arthralgia, traumatic injury, sleeplessness, removing dampness, and relieving itching (Pradeep et al., 2003; Kumar et al., 2014; Nishtha and Thakur, 2021 and references therein). However, little is known about the structure and composition of the ECM community related to this important tree species. Our previous study observed that *C. deodara* harbors less diverse ECM fungi; of 53 ECM fungal species found, only 6 were associated with *C. deodara* (Zhang et al., 2021). Hanif et al. (2012) characterized and identified some ECM species (*Peziza* sp. MHSUC-01, *Russula livescens*, and three species of *Tomentella*) associated with *C. deodara* for the first time using morpho-anatomic and molecular methods targeting its rDNA. To perform some comparative and statistical work in future, we used the same methods for sampling, ECM root-tips collection, molecular identification, and data analysis, as in our previous studies (Wen et al., 2015; Wen et al., 2018; Zhang et al., 2021).

In this research, we assessed the ECM community structure by quantifying and comparing the colonization intensity (abundance and frequency) and diversity of ECM fungal species in urban pure *C. deodara* plantation forests in Nantong City (Jiangsu Province, China), and we characterized soil spore banks by conducting bioassay experiments using these important and useful tree seeds. In addition, we tested soil chemical properties at the different points we sampled. Specifically, our objectives were (1) to characterize whether *C. deodara* has its own preference in ECM fungal associations and to compare the ECM communities between mature trees and soil spore banks at the same site; (2) to characterize abiotic variables and soil properties that may play significant roles in determining ECM fungal communities; and (3) to evaluate the effects of colonizing fungi on seedling growth. Detailed information on ECM fungi associated with *C. deodara* forests is crucial for forest research and planning, and this information can be used for forest growth and ecosystem modeling.

## Materials and methods

### Study sites and sampling

Four isolated points of *C. deodara* forests were conducted in Nantong (Jiangsu Province) in East China: (1) Langshan Park (31°57’ 5” N, 120°53’ 6” E, LSP); (2) Binjiang Park (31°57’ 29” N, 120°52’ 50” E, BJP); (3) the Green Expo Garden (32°1’ 50” N, 120°58’ 23” E, GEG); and (4) a point of the provincial highway (32°2’ 55” N, 121°2’ 49” E, PHW), where the annual average temperature is 15.1°C, and the average rainfall is 1040 mm. Geological and climate information and site conditions are described in **Table 1**. At each point, six to eight *C. deodara* trees were selected for soil samples collection. The distance between any two selected trees should be more than 10 m to ensure the independence of the soil samples. In October 2021, three soil sub-samples (10- × 10- × 20-cm, length × width × depth) were collected within about 1 m from each selected tree and mixed well as 1 sample. In total, 28 soil samples were collected and stored at 4°C until analysis. Then, all roots were picked out from the soil samples, and ECM tips were collected with a root-cutting knife. The ECM samples, placed in an ice box, were transported to the laboratory and stored at 4°C for molecular identification within one week. In total, 4294 ECM root tips (out of 18783 root tips) were collected and placed into 2.0-mL test tubes for DNA analysis.

**Table 1:** Geological, climate information, and soil properties of research sites.

Study sites	LYP	BJP	GEG	PHW	Kruskal–Wallis test	
					*χ2*	*p*
Mean annual temperature (°C)	15.1					
Mean annual precipitation (mm)	1040					
Altitude (m)	10-40	0-10	0-10	0-10		
Latitude/Longitude	N 31°57’ 5”	N 31°57’ 29”	N 32°1’ 50”	N 32°2’ 55”		
	E 120°53’ 6”	E 120°52’ 50”	E 120°58’ 23”	E 121°2’ 49”		
Mean Diameter at Breast Height (cm)	39.63 ± 7.85	33.57 ± 4.28	34.14 ± 6.77	44.67 ± 3.78	9.99	**0.019**
Soil properties						
pH	6.84 ± 0.87	7.27 ± 0.05	7.51 ± 0.12	7.53 ± 0.12	18.539	**< 0.01**
EC(μs/cm)	3.41 ± 1.15	3.38 ± 0.37	2.30 ± 0.89	2.36 ± 1.02	8.539	**0.036**
Available K (mg/kg)	185.33 ± 49.40	172.97 ± 43.09	140.53 ± 21.19	176.38 ± 16.88	6.365	0.095
Available P (mg/kg)	31.77 ± 15.45	14.70 ± 1.66	15.67 ± 4.83	11.01 ± 1.07	17.979	**< 0.01**
Water Soluble Na (g/kg)	14.64 ± 3.86	12.22 ± 2.92	16.31 ± 3.98	18.07 ± 4.65	5.708	0.127
Water Soluble K (g/kg)	20.34 ± 8.93	21.65 ± 4.97	21.27 ± 3.61	22.12 ± 5.20	0.559	0.906
Water Soluble Ca (g/kg)	194.11 ± 64.18	161.97 ± 19.18	152.76 ± 37.69	165.07 ± 44.87	5.351	0.148
Water Soluble Mg (g/kg)	18.73 ± 7.45	18.98 ± 1.40	14.95 ± 4.92	18.82 ± 9.92	2.12	0.548
Total OM(g/kg)	52.16 ± 18.49	42.68 ± 6.95	26.68 ± 11.99	34.42 ± 12.38	10.157	**0.017**

Bold values indicate significant differences (p < 0.05).

After roots collection, all organic debris was carefully removed by hand from the soil samples. Then, it was air-dried indoors, ground, filtered through a 2-mm sieve, and mixed well. Each soil sample was divided into two sub-samples: one for the bioassay experiments and the other for the determination of the chemical properties.

### Bioassay and sampling

Bioassay experiments were performed using an established method (Miyamoto and Nara, 2016; Wen et al., 2018) to assess the soil spore banks of *C. deodara* forests. The bioassay containers were made from 50-mL centrifuge tubes with two drainage holes at the bottom. Each tube was filled with approximately 40 mL of the air-dried soil collected previously. To prevent soil loss, a cotton ball was placed at the bottom of the soil. A total of 84 (3 replicates × 28 soil samples) containers were prepared in total. To check exogenous contamination, 3 negative control containers were used, which were filled with autoclaved soil. Before germination, *C. deodara* seeds were treated according to the method described by Wen et al. (2018). Then, a single germinated seed was placed on the soil surface of each bioassay container and grown at 25°C day/20°C night temperatures under natural light conditions in a glass greenhouse to induce ECM symbionts formation. The containers were watered as needed, and no fertilization was performed.

After 9 months, bioassay seedlings were harvested. All roots and ECM samplings were carefully counted and separated, as described previously. Control seedlings were excluded from further analyses as ECM inoculation was not detected. In total, 5539 ECM root tips (out of 18700 root tips) were separated for molecular analysis. Above- and belowground parts of the bioassay seedlings were dried at 60°C for 72 h and used to determine the dry weight.

### Soil chemical analysis

Soil pH, electric conductance (EC), total organic matter content (OM), available P and K, and water-soluble K, Na, Mg, and Ca were analyzed by standard methods using other soil subsamples.

### Molecular analysis

For molecular analysis, we generally followed the protocols described in Wen et al. (2015; 2018) and Zhang et al. (2021) for DNA extraction, polymerase chain reaction (PCR), and direct sequencing. Internal transcribed spacer (ITS) regions of fungal rDNA were amplified using the ITS 1F and ITS 4 primers. All clean sequences were classified into molecular operational taxonomic units (mOTUs) based on a 97% similarity threshold using the ATGC (GENETYX Corp., Tokyo, Japan) program. After low-quality sequences were discarded, individual mOTUs were compared to sequences in GenBank using Megablast and assigned taxonomic identities. Sequences belonging to known ECM fungal lineages (Tedersoo et al., 2010; Tedersoo and Smith, 2017) were deposited at the NCBI under accession numbers MZ144027–MZ144044 and MZ133282 (**Table 2**), and those that did not were excluded from further analyses.

**Table 2:** Possible identities of ectomycorrhizal fungi formed each ectomycorrhizal type observed in *Cedrus deodara* forests of Nantong in East China.

Species	Acc. Number	R.A^a^		Frequency		Best BLAST Matches		
		Matura	Bioassay	Matura	Bioassay	Description	Ident.	Acc. Number
Ceratobasidium sp	MZ144027	2.18%		2	0	Uncultured fungus clone YJ7 18S ribosomal RNA gene	100.00%	KU931539.1
Geopora sp	MZ144028		2.38%	0	1	Geopora pinyonensis isolate 255 small subunit ribosomal RNA gene	99.65%	MK841899.1
Helotiales sp	MZ144029	1.78%	0.93%	2	1	Uncultured Helotiales clone 1S1.07.F05 18S ribosomal RNA gene	99.59%	EF619697.1
Hydnobolites sp	MZ144030	0.65%		1	0	Uncultured Hydnobolites genes clone: 17	99.31%	LC200533.1
Pezizales sp. 1	MZ144031		14.17%	0	4	Pezizales sp. A14	99.83%	JX434665.1
Pezizales sp.2	MZ144032		2.29%	0	1	Uncultured fungus isolate Q03.13	99.60%	MG274190.1
Pisolithus sp	MZ144033	10.02%		1	0	Pisolithus orientalis isolate LS065 small subunit ribosomal RNA gene	99.52%	MH447973.1
Thelephoraceae sp.1	MZ144034	19.33%		7	0	Uncultured Thelephoraceae genes clone: 1918	98.25%	MN549508.1
Thelephoraceae sp.2	MZ144035	9.44%		2	0	Uncultured ectomycorrhizal fungus genes clone: P09109	96.92%	AB587780.1
Thelephoraceae sp.3	MZ144036		3.01%	0	1	Uncultured Thelephoraceae clone P1_Contig_0347	97.75%	JN704829.1
Tomentella sp.1	MZ144037	0.72%	1.58%	1	1	Uncultured fungus isolate S4NOV.1 small subunit ribosomal RNA gene	98.31%	MK737472.1
Tomentella sp.2	MZ144038	1.64%		1	0	Uncultured fungus clone mOTU49	98.98%	MN549513.1
Tomentella sp.3	MZ144039	0.65%		1	0	Uncultured Tomentella genomic DNA clone Ir84	98.69%	FR852208.1
Tomentella sp.4	MZ144040	0.53%		1	0	Uncultured Tomentella genes clone: 176	99.19%	LC176640.1
Tomentella sp.5	MZ144041	4.26%		2	0	Uncultured fungus ys2 genes	98.69%	LC364196.1
Tomentella sp.6	MZ144042		1.94%	0	1	Uncultured ectomycorrhizal fungus clone Type58	98.19%	HM057221.1
Trichophaea sp	MZ144043	47.75%	6.86%	24	4	Uncultured fungus clone mOTU52	100.00%	MN549516.1
Wilcoxina sp	MZ144044		66.86%	0	24	Wilcoxina mikolae genes strain: TypeA	100.00%	LC029799.1
Pezizaceae sp	MZ133282	1.04%		1	0	Pezizaceae sp. A16	95.62%	JX434666.1

^a^R.A, Relative abundance.

### Data analysis

We used the Kruskal–Wallis test to check the significance of the differences in soil properties among the four sampling sites. For an ECM fungus, the inoculation rates were defined as the percentage of ECM tips colonized by that fungus out of all tips in one soil sample or one bioassay seedling. The frequency was defined as the number of soil samples in which it occurred, and the relative abundance was defined as the percentage of ECM tips colonized by that fungus out of the total number of ECM tips observed. In the bioassay experiments, data from three replicates were combined and treated as an independent sample unit. Soil samples containing no ECM fungal species and sequences not hosted on *C. deodara* were excluded from the following analyses.

To estimate ECM fungal richness and to assess the diversity of soil spore bank communities of *C. deodara* forests, species accumulation curves and Chao2 richness estimators were calculated in the EstimateS program (https://www.robertkcolwell.org/pages/estimates) version 9.1 using 1000 randomizations without replacement, respectively. The species richness, Shannon’s, and Simpson’s diversity indices were calculated for each point both in mature and bioassay experiments. Non-metric multidimensional scaling (NMS), applied *via* PC-ORD version 6 and based on relative Bray–Curtis distance, was performed to visualize ECM fungal communities between mature forests and bioassay experiments. Additionally, redundancy analysis (RDA) was performed to explore the main factors determining the ECM community structure. Data were log + 1 transformed in RDA. A multi-response permutation procedure (MRPP) test was also performed to assess statistical differences in ECM fungal communities between mature forests and bioassay experiments, as well as among the four sites we studied. To determine the significant differences between the seedlings’ growth and the number of ECM fungal species colonizing a seedling, one-way analyses of variance (ANOVAs), with Tukey’s honest significant difference (HSD) tests at p < 0.05, were used. Moreover, linear regression analyses between the seedlings’ growth and inoculation rates of ECM fungi were revealed.

## Results

### Soil properties

Geological and climate information, site conditions, and soil properties are described in **Table 1**. The results of the Kruskal–Wallis test revealed significant differences in the estimated properties among the four sites (**Table 1**). Variation in soil pH among the four investigated sites was small, but the differences were significant (p < 0.01). Samples from site LSP had the highest contents of available P (up to 60.14 mg/kg) and total OM (ranging from 24.49 to 83.20 g/kg), whereas the soil pH (ranging from 4.86 to 7.44) in these samples was lower. Nevertheless, samples from site GEG had the lowest total OM (26.68 ± 11.99 g/kg), and site PHW had the lowest content of available P (11.01 ± 1.07 mg/kg) (**Table 1**). Lower pH and total OM content favor the formation of mycorrhizae (Zhang et al., 2021; Wen et al., 2022).

### ECM fungal species summary

#### Mature forests

Of the 28 soil samples, 26 contained ECM root tips, but only 13 ECM fungal species were identified. At the species level, *Trichophaea* sp.1 (48%) and Thelephoraceae sp.1 (19%) were dominant on *C. deodara* and were found in 24 and 7 soil samples, respectively. Only 2 ECM fungi were identified in more than 5 soil samples. On the contrary, 7 ECM fungal species were singletons, which were identified in a single sample (**Table 2**).

#### Bioassay experiments

Most bioassay seedlings (82%) were alive at the end of the 9-month experiment. *Wilcoxina* sp. was the most dominant on *C. deodara* bioassay seedlings and found in 24 soil samples. In total, ECM root tips formed on the seedlings in 26 soil samples. Moreover, up to 3 ECM species were found in individual soil sample, and only 1 or 2 ECM species were found in the majority of soil samples (**Table 2**).

**Figure 1:**
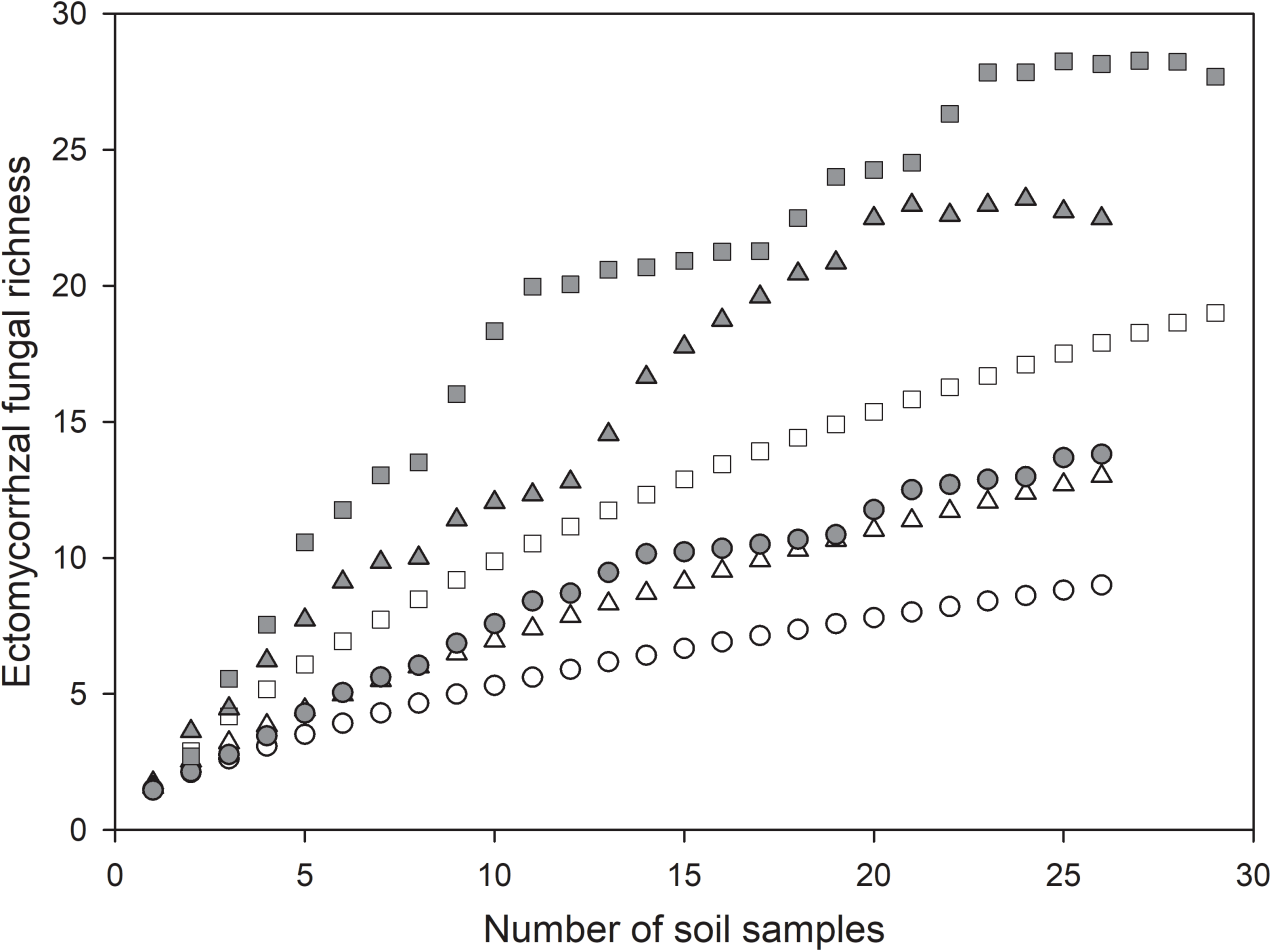
Species accumulation curves for ectomycorrhizal (ECM) fungi found in *Cedrus deodara* forests. Circles, triangles, and squares represent bioassay, mature, and total observed ECM fungal species richness (open) and Chao2 minimum species richness estimates (filled), respectively.

In all, we identified 19 ECM fungal species, of which 13 species were found in mature forests and 9 species were detected in bioassay experiments, while only 3 species were shared between mature forests and bioassay seedlings. The chao2 (± SD) richness estimator indicated that at least 28± 9 ECM fungal species were expected to inhabit these forests; meanwhile, the estimators for mature forests and bioassay experiments were 23 ± 9 and 14 ± 6, respectively (**Figure 1**). In sites LYP and BJP, both the Shannon’s and Simpson’s diversity values were higher in resident ECM communities in mature trees than in the spore banks. However, the other two sampling sites (GEG and PHW) showed a completely contradictory conclusion (**Table 2**). In addition, the curves Chao2 (filled) minimum species richness estimates of ECM fungi (**Figure 1**) reached their asymptote for the overall dataset. Additional ECM species would be found with more soil samples collecting.

### ECM fungal species community structure

**Figure 2:**
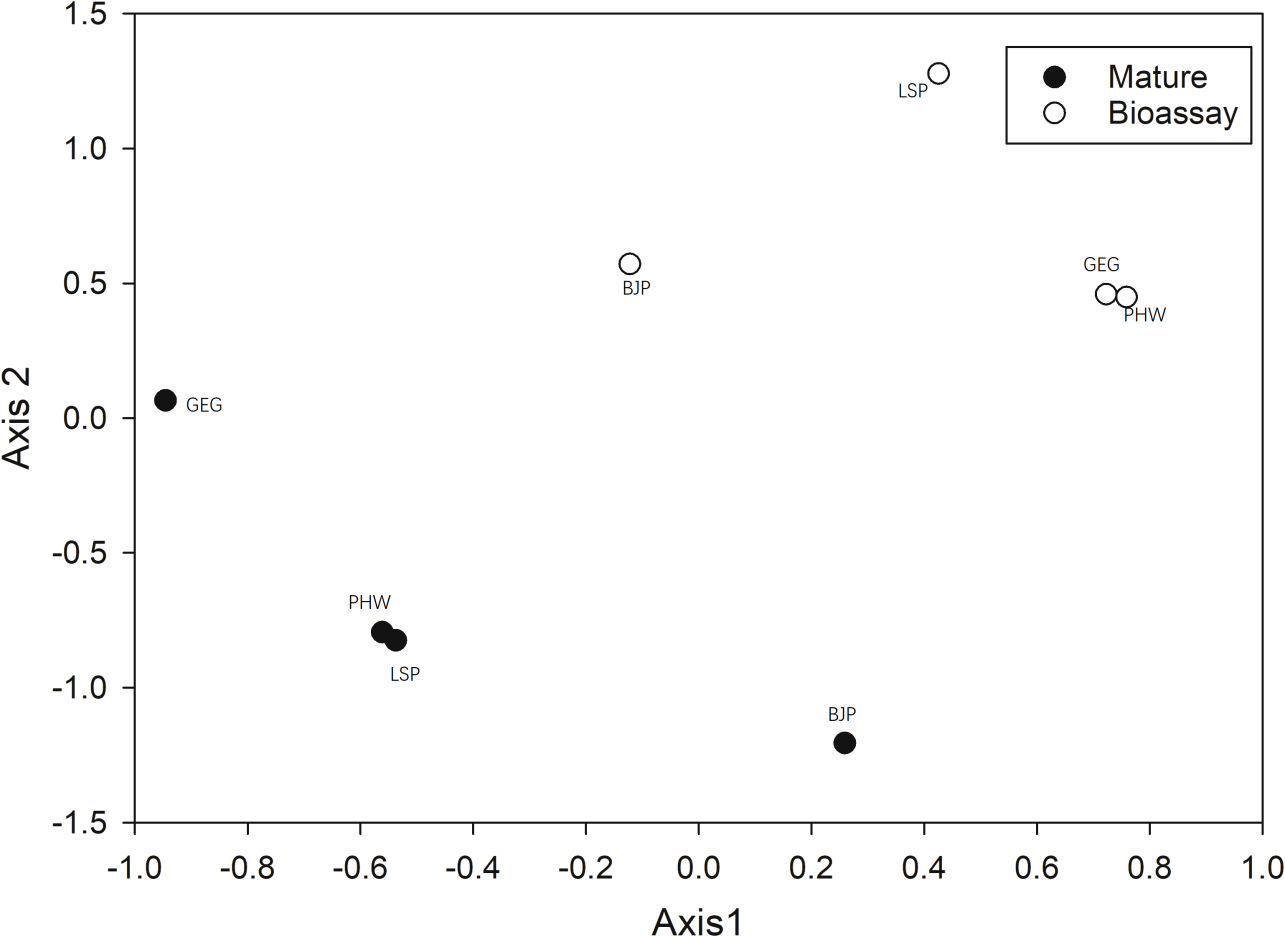
Non-metric multidimensional scaling (NMS) of ectomycorrhizal (ECM) fungal communities in resident *Cedrus deodara* tree roots (Mature) and soil spore banks (Bioassay), based on the relative Sørensen distance. LSP, Langshan Park; BJP, Binjiang Park; GEG, Green Expo Garden; PHW, Provincial highway.

The NMS ordination, based on the relative Bray–Curtis distance, clearly separated ECM fungal communities between mature forests and spore banks (**Figure 2**). The MRPP test also confirmed this statement (A = 0.391, p = 0.006). In contrast, site had no significant effect on ECM fungal community composition in either mature forests (A = 0.088, p = 0.066) or spore banks (A = 0.043, p = 0.081).

**Figure 3:**
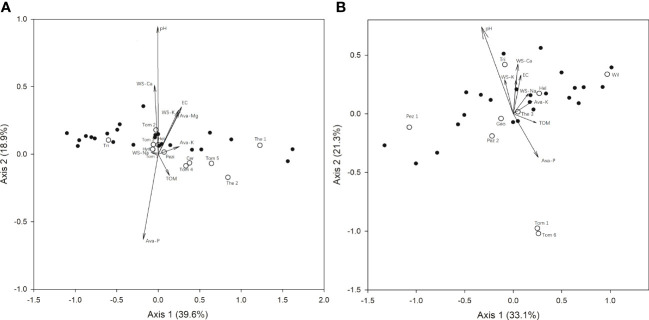
Redundancy analysis (RDA) of ectomycorrhizal fungi detected [**(A)** mature forests and **(B)** soil spore banks] in important *Cedrus deodara* forests with reference to soil parameters. Ava, Available; WS, Water Soluble; TOM, Total Organic Matter; Cer, *Ceratobasidium*; Geo, *Geopora*; Hel, Helotiales; Hyd, Hydnobolites; Pez, Pezizales; Pis, *Pisolithus*; The, Thelephoraceae; Tom, *Tomentella*; Tri, *Trichophaea*; Wil, *Wilcoxina*; Pezi, Pezizaceae. Species names are based on **Table 2**.

In mature forests, RDA showed that ECM fungal communities were significantly correlated with soil properties. The eigenvalues of Axes 1 and 2 were 0.88 and 0.27 in the RDA biplots (**Figure 3A**), explaining 39.6% and 12.9% of the total variance in the community data, respectively. Axis 2 had a marked correlation with pH (r = 0.94) and available P content (r = -0.63). Meanwhile, in spore banks, the eigenvalues of Axes 1 and 2 were 0.59 and 0.29 in the RDA biplots (**Figure 3B**), explaining 33.1% and 21.3% of the variance in the community data, respectively. Axis 2 had a markedly positive correlation with pH (r = 0.81), indicating that ECM fungal communities were significantly correlated with soil pH. In addition, *Tomentella* sp.1, *Tomentella* sp.6 (in spore bank), and *Pisolithus* sp. (in mature) occurred in soils with a low pH and high available P content, while *Trichophaea* sp. occurred at a high pH.

### Effects of ECM fungi on the growth of bioassay seedlings

**Figure 4:**
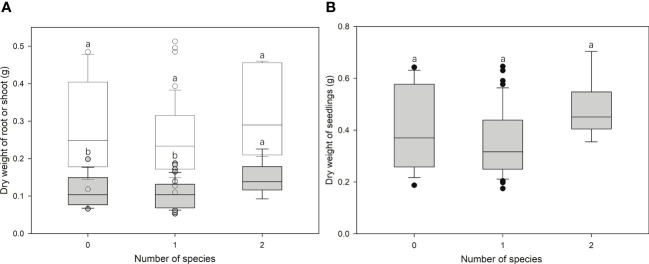
Correlation between the growth of bioassay *Cedrus deodara* seedlings [**(A)** dry weight of root (filled) or shoot (open); **(B)** dry weight of total seedlings] and ectomycorrhizal fungal richness colonized from soil spore banks in *Cedrus deodara* forests. Only one seedling was colonized with three ECM fungal species and was excluded from analyses.

**Figure 5:**
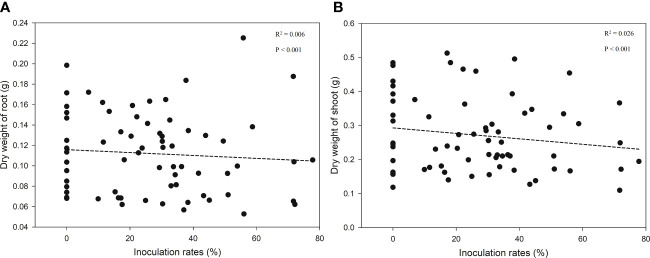
Regression between seedlings growth [**(A)** dry weight of root; **(B)** dry weight of shoot] and inoculation rate.

ECM richness had significant effects (F = 3.028, p < 0.05) on the root growth of bioassay seedlings in ANOVA but had no effects on the shoot (F = 1.778, p = 0.177) or total (Root + Shoot) biomass (F = 2.188, p = 0.120) (**Figures 4A, B**). In addition, linear regression analyses revealed no effect of inoculation rates on seedlings growth (**Figures 5A, B**).

## Discussion

To the best of our knowledge, this investigation represents the first documentation of ECM fungi that can form ECM symbionts with *C. deodara* in forests and bioassay experiments. In general, host specificity, evolution and diversity, geographic distribution, and ecology systems can influence ECM fungal communities (Tedersoo and Smith, 2013). Belowground ECM fungal communities in *C. deodara* forests showed a typical and special structure, including a few abundant and plenty of rare species. Accordingly, the sporocarp investigation carried out by Itoo and Reshi (2014) in India showed similar results. Of the 44 ECM species associated with *C. deodara*, 24 were rare. However, we cannot underestimate the function of rare species in forest dynamics. The spatial mechanism of assembling ECM fungal communities remains unclear, particularly in coastal areas where such investigations are scarce.

The diversity and community structure of ECM fungi are essential for the establishment of coastal pine forests. Some fungal species belonging to Thelephoracae (e.g., Thelephoracae spp. and *Tomentella* spp.), the richest family in this study, were detected in both resident trees and soil spore banks, as the results showed. They have also been observed to be the dominant species in coastal pine forests (Kataoka et al., 2008; Matsuda et al., 2009; Zhang et al., 2021). *Trichophaea* spp. have been observed to be the first or third most dominant species in mature tree roots or soil spore banks. Sun et al. (2020) found that *Trichophaea* play an important role in obtaining nutrients from saline soil, suggesting that such ECM fungi may have a priority in colonizing *C. deodara* along coastal lines. *Wilcoxina* sp. was the most commonly detected ECM fungus in *C. deodara* seedlings in the bioassay experiments (**Table 3**). Consistent with this, this mycorrhizal symbiont was typically dominant in 1-year-old host seedlings (Rudawska and Leski, 2021) and forest nursery seedlings (Klavina et al., 2016). *Wilcoxina* spp. are dominant in sandy lands (Guo et al., 2020). They can improve the survival and tolerance of their hosts to salinity stresses (Zwiazek et al., 2019). Interestingly, they were not found in mature trees during this study. We hypothesized that *Wilcoxina* was the major genus, but its abundance decreased with an increase in stand age. In addition, ECM fungal communities commonly differ in the ability of their dispersal and fungal propagules to disperse in new habitats (Kennedy and Bruns, 2005; Kennedy, 2010). This agrees with previous findings (Wu et al., 2018; Wang et al., 2019) in root-associated ECM fungal communities. However, evidence of this and its potential effects on seedling performance is scarce (Smaill and Walbert, 2013).

**Table 3:** Comparison of ectomycorrhizal fungal diversity between mature forests and soil spore banks in *Cedrus deodara* forests of Nantong in East China.

	Name	S	E	H	D`
Mature forests	LYP	4	0.747	1.035	0.5973
	BJP	7	0.811	1.579	0.7496
	GEG	4	0.378	0.524	0.2361
	PHW	3	0.655	0.720	0.3969
Bioassay experiments	LYP	4	0.461	0.639	0.2998
	BJP	4	0.707	0.981	0.5116
	GEG	3	0.745	0.818	0.5324
	PHW	4	0.713	0.989	0.5194

Richness (S) and estimated species richness (E, Evenness; H′, Shannon’s diversity index; D′ Simpson’s diversity index)

Most ECM fungal genera have a global distribution, while their hosts do not (Tedersoo and Nara, 2010), meaning that ECM fungal communities may vary among host plants in different habitats. Globally, the ECM fungal community structure is significantly influenced by the host, geographic position, soil properties, and climatic variables, of which the host may be the most significant factor (Ishida et al., 2007; Smith et al., 2009; Bahram et al., 2012; Tedersoo et al., 2012; Põlme et al., 2013; Roy et al., 2013; Velmala et al., 2013; Miyamoto et al., 2014). Therefore, to explore the influence of abiotic factors, the most effective way to eliminate host effects is to focus on only one host species and investigate the coexistence mechanism of a single species.

Here, we carried out research on the community and structure of ECM fungi in pure *C. deodara* plantation forests. Consistent with our previous work (Wen et al., 2015; Wen et al., 2018; Zhang et al., 2021), we found that at large scales, the site was the overriding factor affecting the structure of the ECM fungal community. However, on local scales, as the results showed, the ECM fungal communities did not differ significantly among the four sampling sites in this study system. We hypothesized that the strength of selection and biotic interactions decreases at the smallest scale as the environment becomes more homogeneous (Vályi et al., 2016; Prieto−Rubio et al., 2022), causing similarity in the ECM community structure. However, in the present study, ECM fungal communities on mature trees and spore bank communities were well documented to be related to soil characteristics (Smith and Read, 2008; Dickie et al., 2009; Alzetta et al., 2012; Glassman et al., 2015; Miyamoto and Nara, 2016; Wen et al., 2018; Zhang et al., 2021). Here, we confirmed that soil pH and available P content had significant effects on ECM species occurrence in both mature trees and young seedlings (**Figure 3**) as ECM communities exhibit complex structural and functional responses to the surrounding environment. In addition, the RDA analysis showed that the soil properties we tested here only collectively accounted for 52.5% and 54.4% of the total variation in mature forests and bioassay seedlings, respectively. Accordingly, we supposed that the local process, except for the tested soil parameters, includes species interaction, habitat filtering, and dispersal limitation, which might determine the abundance of coexisting species on local scales. Our study contributes to the increasing literature demonstrating the effect of the environment on the fungi.

Except for richness effects on roots, ECM fungal inoculation rates did not cause any difference in host seedling growth (**Figures 4** and **5**) as adequate soil nutrient levels might mask the contribution of ECM fungi to the host. These results were confirmed by the studies of Makita et al. (2012) and Kayama and Yamanaka (2014), where there was no significant difference in the dry weight of seedlings inoculated with different ECM fungal species. The promotion of plant growth by ECM symbionts has been widely reviewed (Marschner, 2012). Moreover, findings concerning the high and low biomass of host seedlings were found for the relative abundances of ECM fungi. Only being colonized with specific ECM species, such as *Suillus* and *Rhizopogon*, could improve seedling growth, rather than colonization with other species (Qu et al., 2003; Sousa et al., 2011; Murata et al., 2017; Wen et al., 2018; Castaño et al., 2023). This means that the effects of ECM fungi on host plant growth differ among species due to their different physiological traits (Onwuchekwa et al., 2014; Peay and Bruns, 2014; Wen et al., 2018).

In summary, although this study conducted a limited sampling effort (four sites with 28 soil samples) and came with the limitations of studying symbionts in a greenhouse under artificial conditions, our findings suggest that: (1) soil pH and available P content, with another local process, had significant effects on species occurrence both in mature trees and young seedlings, with ECM communities clearly separated from each other on local scales; (2) *C. deodara* might have its own preference in association with Thelephoracae, the richest family we detected; and (3) the promotion of ECM symbionts to plant growth has been widely confirmed but only when colonized with specific ECM species, and the effects of ECM fungi on host plant growth differ among different ECM fungal species. Accordingly, these findings are worth further investigation with more sampling sites, and to conserve this important tree species, we should take ECM fungi that are associated with it into consideration.

## Data availability statement

The datasets presented in this study can be found in online repositories. The names of the repository/repositories and accession number(s) can be found in the article/supplementary material.

## Author contributions

LS and BT designed the study. SM and YP performed the bioassay experiments. ZW analyzed the data and wrote the manuscript. ZW, CL, and XX conducted the field sampling work. YS gave some suggestions for language improvement. LS revised the final manuscript. All authors contributed to the article and approved the submitted version.
